# Efficacy and safety of pharmacological treatments for neuroborreliosis—protocol for a systematic review

**DOI:** 10.1186/2046-4053-3-117

**Published:** 2014-10-21

**Authors:** Rick Dersch, Michael H Freitag, Stefanie Schmidt, Harriet Sommer, Gerta Rücker, Sebastian Rauer, Joerg J Meerpohl

**Affiliations:** 1 German Cochrane Centre, Medical Center–University of Freiburg, Berliner Allee 29, 79110 Freiburg, Germany; 2 Department of Neurology, Medical Center–University of Freiburg, Breisacher Str. 64, 79104 Freiburg, Germany; 3 Institute of General Practice and Family Medicine, Jena University Hospital, Friedrich-Schiller-University, Bachstr. 18, D-07743 Jena, Germany; 4 German Society for Urology, Kuno-Fischer-Strasse 8, 14057 Berlin, Germany; 5 Institute of Medical Biometry and Statistics, Medical Center–University of Freiburg, Stefan-Meier-Str. 26, 79104 Freiburg, Germany

**Keywords:** Neuroborreliosis, Lyme disease, Meta-analysis, Non-randomized studies, Drug treatment, Systematic review, Decision-making, Randomized controlled trials

## Abstract

**Background:**

Neuroborreliosis is a tick-borne infectious disease of the nervous system caused by *Borrelia burgdorferi*. Common clinical manifestations of neuroborreliosis are cranial nerve dysfunctions, polyradiculoneuritis, and meningitis. Diagnosis is usually based on clinical presentation, serologic testing, and analysis of cerebrospinal fluid. Many aspects of pharmacological treatment, such as choice of drug, dosage, and duration are subject of intense debate, leading to uncertainties in patients and healthcare providers alike. To approach the questions regarding pharmacological treatment of neuroborreliosis, we will perform a systematic review.

**Methods:**

We will perform a comprehensive systematic literature search for potentially eligible studies that report outcomes after pharmacological interventions. To adequately consider the wealth of research that has been conducted so far, this review will evaluate randomized controlled trials (RCTs) and non-randomized studies on treatment of neuroborreliosis. We will assess potential risk of bias for each RCT meeting our selection criteria using the Cochrane risk of bias tool for RCTs. For non-randomized studies, we will use the Newcastle-Ottawa Scale and the recently piloted Cochrane risk of bias tool for non-randomized studies. Our primary outcome of interest will be neurological symptoms and the secondary outcomes will be disability, patient-reported outcomes (quality of life, and, if reported separately from other neurological symptoms, pain, fatigue, depression, cognition, and sleep), adverse events, and cerebrospinal fluid pleocytosis. Pooling of data and meta-analysis will only be deemed justified between studies with similar design (e.g., RCTs are only combined with other RCTs), characteristics (e.g., similar populations), and of acceptable heterogeneity (*I*^2^ < 80%). Pooled estimates will be calculated using RevMan software. Prespecified subgroup analyses will evaluate groups of antibiotics, length of antibiotic treatment, and different doses of doxycycline. We will assess the quality of evidence using the Grading of Recommendations Assessment, Development and Evaluation (GRADE) approach.

**Discussion:**

This systematic review will summarize the available evidence from RCTs and non-randomized studies regarding pharmacological treatment of neuroborreliosis. The available evidence will be summarized and discussed to provide a basis for decision-making for patients and healthcare professionals.

**Systematic review registration:**

PROSPERO registration number: CRD42014008839

## Background

Lyme neuroborreliosis is a tick-borne infectious disease caused by the gram-negative spirochete bacterium *Borrelia burgdorferi sensu lato*. Transmission of *B. burgdorferi* occurs via bites by the tick species Ixodes ricinus in Europe and Ixodes scapularis and Ixodes pacificus in the United States. Different species of *B. burgdorferi* show distinct patterns of distribution, whereas *Borrelia garinii, B. afzelii*, and *B. burgdorferi sensu stricto* are common in Europe; in the USA, *B. burgdorferi sensu stricto* predominates [1]. The different genospecies seem to be associated with distinct clinical manifestations, i.e., *B. garinii* being more closely associated with neurological manifestations of borreliosis than other species [1]. Affection of the nervous system occurs in approximately 15% of all patients with lyme borreliosis [2]. Common clinical manifestations of neuroborreliosis are polyradiculoneuritis (Bannwarth’s syndrome, 63.75% of all cases), in 60% accompanied by cranial nerve dysfunctions (most frequently facial palsy), and meningitis (23.75%) [3,4]. To a lesser extent, affections of the central nervous system like encephalitis and myelitis occur (12.5%) [4]. Other, rarer manifestations are borrelia-induced vasculitis (0.3%) and myositis (case reports) [5-8]. Beside nervous system affections, *B. burgdorferi sensu lato* can affect multiple other organ systems. In Europe, manifestations include the early developing erythema migrans (89%), acrodermatitis atrophicans (1%), lyme arthritis (5%), or lyme carditis (<1%) [1,9]. Diagnosis of neuroborreliosis is usually based on clinical presentation, serologic testing, and analysis of cerebrospinal fluid (CSF) [10]. Tiered case definitions exist regarding likelihood of diagnosis depending on diagnostic results [3,11]. Different authors report variable sensitivities of this approach, and other diagnostic strategies of unclear accuracy have been suggested [12-14]. Controversy exists also on the field of therapy, where choice, route of administration, and length of treatment are subject of intense debate. The guidelines of the Infectious Diseases Society of America (IDSA), the European Federation of Neurological Societies (EFNS), and the evidence-based practice parameters of the American Academy of Neurology (AAN) recommend antibiotic treatment with a duration of up to 21–28 days [10,15,16], whereas guidelines of the International Lyme and Associated Diseases Society (ILADS) states that several months of antibiotic therapy are often required [17]. Furthermore, the ILADS guideline states that short-course antibiotic treatment shows high failure rates for treatment of neuroborreliosis. Choice of antibiotic agent for treatment of neuroborreliosis is also a matter of ongoing controversy. IDSA, EFNS, and AAN guidelines usually recommend cephalosporin antibiotics, doxycycline, or penicillin antibiotics, whereas some authors recommend treatment with other substances, like carbapenem antibiotics, metronidazole, or antimalarial drugs such as hydroxychloroquine for certain subgroups of patients [17]. The use of combination therapy (use of multiple antibiotics concurrently) for the treatment of neuroborreliosis is advocated by the ILADS, whereas the IDSA, EFNS, and AAN discourage the use of antibiotic combination regimes. The optimal dosage of the orally administered antibiotic doxycycline remains unclear. Studies investigating the effects of doxycycline on people with neuroborreliosis examined daily doses ranging from 200–400 mg [18-20]. Accordingly, the IDSA and EFNS guidelines recommend doxycycline doses of 200 mg, whereas the guidelines of the Deutsche Gesellschaft für Neurologie (DGN) and AAN guidelines suggest applying higher doses of up to 400 mg [10,15,16,21]. These various, partly contradicting recommendations lead to a considerable ambiguity and doubt in patients and healthcare providers alike when facing treatment decisions for neuroborreliosis. Therefore, it seems necessary to review, evaluate, and summarize the available evidence for drug treatment of neuroborreliosis for making evidence-based clinical recommendations. To adequately consider the wealth of research that has been conducted and to overcome limitations of availability of only few RCTs for a limited number of important questions with regard to optimal treatment, this review will evaluate randomized controlled trials and non-randomized studies for treatment of neuroborreliosis. Adding non-randomized studies to systematic reviews can have certain advantages. Non-randomized studies seem to be more suitable for the detection of adverse events than RCTs and may have longer follow-up periods [22].

## Method/design

### Objectives

To assess the efficacy and safety of pharmacological interventions in the treatment of adults with neuroborreliosis.

### Types of studies

This review will evaluate randomized controlled trials and non-randomized studies for treatment of neuroborreliosis. By assessing randomized and non-randomized studies, we will consider the wealth of research that has been conducted and overcome scarcity and limitation in scope of available RCTs. All studies comparing any pharmacological treatment to any other treatment, placebo, or no treatment will be screened for eligibility. If studies report no comparison group for an intervention, their results will only be reported narratively in an appendix to capture the available literature. Studies without control group will not be used to calculate pooled estimates and will not be subject to a separate risk of bias assessment. Single case reports will not be reviewed, but case series greater than five patients will be considered.

### Types of participants

Studies which evaluate adults with clinically diagnosed neuroborreliosis will be included. Diagnosis of neuroborreliosis is based upon the clinical case definition by Halperin and Kaiser [3,11]. This case definition distinguishes between ‘possible’, ‘probable’, and ‘definite’ neuroborreliosis describing an increased likelihood of neuroborreliosis based on clinical and laboratory parameters. For the diagnosis of ‘possible’ neuroborreliosis, the presence of neurological symptoms suggestive of lyme neuroborreliosis without other obvious reasons are required, additionally to one of the following criteria: borrelia antibodies in serum, CSF pleocytosis, intrathecal synthesis of borrelia-specific antibodies, or a verified erythema migrans during the past 4 months. Serology should be performed in a two-staged approach, the first step being a sensitive ELISA and the second a more specific immunoblot [3,11,23]. For ‘probable’ neuroborreliosis, a CSF analysis with pleocytosis and intrathecal production of IgG is mandatory. For the diagnosis of ‘definite’ neuroborreliosis, additional positive intrathecal synthesis of borrelia-specific antibodies is mandatory. Positive findings for PCR or culture of *B. burgdorferi* in CSF are supportive of the diagnosis but have a high false negative rate. Studies regarding patients with ‘post-lyme disease’, defined as people with persistent symptoms in the absence of evidence for ongoing infection, will be excluded.

### Types of interventions

We will consider any pharmacological treatment, including combinations of treatments. Main interventions which we are expecting are the following: antibiotics, steroids, analgesic agents, and phytotherapeutics. We plan to compare any of these interventions with placebo/no intervention or any other of these types of interventions separately. Both for antibiotics and steroids, it will be reported if drug dosage was adjusted to body weight.

### Types of outcomes

#### Primary outcomes

1. Neurological symptoms after treatment. Neurological symptoms should be measured by a validated method (either assessed by a patient or clinical staff, e.g., via the Neurological Impairment Scale (NIS) or in analogy to the Expanded Disability Status Scale (EDSS) [24,25]). When information is lacking or no valid method was used, which is assumed to be the case in the majority of studies, neurological symptoms will be considered as defined by the original authors of the study. If several time points are reported in a primary study, data from the last reported time point will be considered. If data permits, results will be presented for short term follow-up (1 to 3 months following the start of treatment) and for long term follow-up (4 or more months following the start of treatment). If multiple time points are reported for short- or long-term outcome, we will consider the latest time point reported. If data permits, we will interpret neurological symptoms as a continuous outcome using post-intervention scores. Lack of validated measurement of outcomes will be considered in the risk of bias assessment and robustness of data evaluated in a sensitivity analysis.

#### Secondary outcomes

1. Adverse events. We will consider any adverse event as defined and reported by the original authors. Adverse events will be reported as serious adverse events when they require hospitalization, are life-threatening or fatal.

2. Overall disability after treatment at short term follow-up (1 to 3 months following the start of treatment) and at long term follow-up (4 or more months following the start of treatment). Disability should be measured with a validated scale, e.g., the modified Rankin scale [26]. If several time points are reported, data from the last time point will be considered. If data permits, results will be presented for short term follow-up (1 to 3 months following start of treatment) and for long term follow-up (4 or more months following start of treatment). Disability will be treated as a continuous outcome. If other validated scales were used by the original authors, measurements of disability will be considered as stated by the authors.

3. Measures of patient reported outcomes, as long as they are measured by a validated scale, at 1 to 3 months and at 4 or more months following the start of treatment. This will be regarded as a continuous outcome. The main patient reported outcome of interest is health-related quality of life. Other relevant patient reported outcomes that will be reported, if assessed separately from other neurological symptoms and quality of life, will be pain, fatigue, depression, cognition, and sleep. Improvement will be considered according to the minimum important difference (MID), if available, of each used validated scale. When no validated scale was used to provide outcome data, improvement will be considered as stated by the original authors.

4. Cerebrospinal fluid pleocytosis after treatment. If several time points are reported in a primary study, data from the last reported time point will be considered. This will be treated as a continuous outcome.

### Search methods for identification of studies

#### Electronic searches

We will search the databases MEDLINE (via Ovid, from 1950 to the present), EMBASE (via Scopus, from 1980 to the present), and the Cochrane Central Register of Controlled Trials for eligible studies. Search strategies are shown in Appendix 1, 2 and 3. No language restrictions are set.

### Searching for other resources

We will also search three trial registers (http://www.controlled-trials.com, http://www.clinicaltrials.gov, http://www.who.int/trialsearch/) to identify additional published or unpublished studies or data for completed studies as well as ongoing studies. The reference list of included studies will be screened for further eligible studies.

### Data collection and analysis

#### Selection of studies

Firstly, one reviewer will evaluate the titles and abstracts to determine whether the study meets the eligibility criteria. Secondly, full texts will be evaluated independently by two reviewers for eligibility. Disagreements will be resolved by a discussion with a third reviewer.

### Data extraction and management

Two review authors will independently extract data from the full texts of included studies using a specifically developed extraction form. The data extraction form will be piloted previously.

Information will be collected on the following:

1. study characteristics (first author, geographical origin, year of publication, start and end of study, study design, number of arms, sample size, duration of follow-up)

2. participant characteristics (age, sex, numbers of participants, how diagnosis was performed, case definitions, disease manifestations, inclusion and exclusion criteria in the included studies, baseline imbalances between study arms and possible confounders (disease manifestation, delay between onset of symptoms and treatment, previous treatment, co-medication, co-morbidities and other confounders as reported by the authors)

3. intervention and comparator details (sample size for each treatment arm, blinding, dose and type of interventions, dosage adjustment based on body weight, duration of treatment, withdrawals and drop-outs)

4. outcome measures (description of measurement tools used, data for continuous/dichotomous/categorical efficacy variables, time point of measurement, adverse events and serious adverse events).

When adjusted analyses are available in primary studies, these adjusted estimates of treatment effects will be used. Otherwise, we will extract the unadjusted data as reported in the primary study. This fact will be considered accordingly in the risk of bias assessment and will be subject to sensitivity analyses. Data will be entered into Review Manager (RevMan 5.3) by one of the reviewers and checked by a second reviewer. Discrepancies in data extraction or entry will be resolved by a discussion with a third reviewer. Reviewers will not be blinded to study author, journal, or institution.

### Assessment of risk of bias of included studies

The assessment of risk of bias will be performed by two reviewers independently considering the following domains according to the Cochrane risk of bias tool: sequence generation, allocation concealment, blinding (of participants, personnel, and outcome assessors), incomplete outcome data, selective outcome reporting, and other sources of bias for the RCTs. According to the Cochrane Handbook, these items will be described as having a ‘low’, ‘high’, or ‘unclear’ risk of bias [27]. For non-randomized studies, bias due to confounding, bias in selection of participants, bias due to departures from intended interventions, bias due to missing data, bias in measurements of outcomes of interventions, bias in selection of the reported results, and overall bias will be assessed according to the ‘Cochrane risk of bias tool for non-randomized studies’ [28]. According to the ‘Cochrane risk of bias tool for non-randomized studies’ these items will be described as having a ‘low’, ‘moderate’, ‘serious’, ‘critical’, or ‘unclear’ risk of bias. At the time of writing this protocol, the ‘Cochrane risk of bias tool for non-randomized studies’ (RoB) is currently being piloted. Therefore, additionally, the Newcastle-Ottawa Scale (NOS) will be used to assess risk of bias and methodological quality [29]. We expect that the major confounders which could influence effect measures in neuroborreliosis are ‘time from onset of symptoms until treatment’ and ‘age’. ‘Time from onset of symptoms until treatment’ is included in the NOS-rating scale as an assessment factor for ‘comparability’ and is regarded as a major criterion for the domain ‘bias due to confounding’ in the ‘Cochrane RoB tool for non-randomized studies’. Non-randomized studies will be classified as having a ‘high risk’ of bias when having an assessment as being of ‘serious’ risk in the Cochrane RoB-tool or when having less than four stars on the NOS-rating scale. In the event of disagreement, consensus will be achieved through a discussion with all review authors. According to the recommendations for the Cochrane RoB-tool for non-randomized studies, no studies assessed as having a ‘critical’ risk of bias will be included in any data synthesis.

### Measures of treatment effect

We will analyze the primary outcome ‘neurological symptoms’ as a continuous outcome. If more than one scale is used to measure outcomes in the same study, only validated scales will be considered. In the case when more than one validated or more than one but only unvalidated scales are reported for one outcome, we will use the results provided by the scale which is mostly used in the other included studies. If data does not permit analysis of ‘neurological symptoms’ as a continuous outcome, we will use the reported data from primary studies and treat this outcome as a dichotomous outcome ‘presence of neurological symptoms’. The treatment effect for each continuous outcome (neurological symptoms, disability, patient reported outcomes, and cerebral fluid pleocytosis) will be expressed as a mean difference (MD) with 95% confidence interval (CI). Where continuous outcomes are measured using different scales, the treatment effect will be expressed as a standardized mean difference (SMD) with 95% CI. As recommended by Guyatt et al., when possible, the treatment effects will be additionally expressed by the ratio of means (RoM) with 95% CI to facilitate interpretation [30,31]. The treatment effect for dichotomous outcomes (adverse events) will be expressed as a risk ratio (RR) with 95% confidence intervals.

### Unit of analysis

The unit of analysis is each patient recruited in the studies.

### Dealing with missing data

Data will be analyzed on an intention-to-treat basis whenever possible. If data are only available in graphical format, we will thoroughly estimate the numerical values.

### Assessment of heterogeneity

Heterogeneity among studies will be investigated by using the chi^2^ test and *I*^2^ test. If significant heterogeneity is detected (*I*^2^ > 50% or *p* <0.1) for outcome measures, the calculations with a fixed effect model will be repeated using a random effects model as sensitivity analysis and we will consider results from both.

### Assessment of reporting biases

We plan to minimize the impact of reporting bias in our systematic review by ensuring a comprehensive search for eligible studies including three trial registries. A funnel plot and appropriate statistical tests for small study effects will be performed if ≥10 studies are available [32].

### Data synthesis

Intervention effects in divergent study designs are influenced differently by bias. Data from RCTs and non-randomized studies will not be pooled, but will rather be analyzed separately. Combined estimates will not be provided for studies with considerable imbalances or differences in the included population or differences regarding interventions. Estimation of treatment effects will be based on a fixed effect model; when we are faced with substantial heterogeneity (i.e., *I*^2^ > 50%), a random effects model will be calculated as well as sensitivity analysis. Pooling of data and meta-analysis of non-randomized studies will only be considered among studies with similar design (e.g., prospective cohort studies will only be combined with other prospective cohort studies) and limited heterogeneity. We will calculate pooled RRs and 95% CIs across comparable studies using Review Manager (RevMan 5.3). When considerable heterogeneity (*I*^2^ > 80%) is found between comparable studies, pooled estimates will not be provided. Instead, a descriptive synthesis of findings will be performed.

### Subgroup analysis and investigation of heterogeneity

We plan subgroup analyses to evaluate effects of different lengths of antibiotic treatment regardless of antibiotic group within prespecified durations (up to 14 days, 14–28 days and longer than 28 days). Due to the clinical importance of antibiotic treatment options, subgroup analyses focussing on different antibiotics are of considerable interest. We will evaluate prespecified classes of antibiotics (doxycycline, cephalosporins and penicillines, other antibiotics and combinations of antibiotics) in subgroup analyses. Cephalosporins and penicillines will be lumped together as beta-lactam antibiotics to be compared to other groups of antibiotics. As for the antibiotic agent doxycycline, the adequate dosage is of particular relevance, a subgroup analysis will be performed comparing daily doxycycline doses ≤200 mg with doses >200 mg.

Differences in diagnostic likelihood of case definitions can lead to heterogeneity due to differences in the study population. The differences between the case definitions of ‘probable’ and ‘definite’ neuroborreliosis are marginal, and it is likely that they represent the same population. In contrast, the case definition of ‘possible’ neuroborreliosis has broader inclusion criteria and may contain patients with other conditions accidentally and therefore may introduce a different population. A subgroup analysis will be performed to investigate whether treatment effects differ in relation to the case definitions used in the primary studies. As clinical likelihood of diagnosis in ‘probable’ and ‘definite’ neuroborreliosis is very similar, we will merge these two case definitions to be compared with ‘possible’ neuroborreliosis cases.

The variety of causative organisms in Europe compared to the United States could lead to heterogeneity, consequently the effect of geographical origin of the study population will be evaluated in a subgroup analysis.

### Sensitivity analysis

We plan to test the robustness of the results by repeating the analysis using a random effects model when confronted with substantial heterogeneity. Sensitivity analyses will also be conducted to assess the effect of risk of bias in the included studies; these sensitivity analyses will be conducted for both risk of bias assessments using NOS and the Cochrane NRS tool. If studies with adjusted and unadjusted data are pooled, the effect of the studies with unadjusted data will also be subject to sensitivity analyses. If data permits, we will perform a sensitivity analysis treating the outcome ‘neurological symptoms’ as a categorical outcome using a proportional odds model to assess the robustness of results [33].

### Assessing the quality of evidence

We will use the Grading of Recommendations Assessment, Development and Evaluation (GRADE) approach to assess the quality of evidence for each outcome. We will judge the quality of evidence based on the suggested five criteria for downrating our confidence in effects estimates (risk of bias, inconsistency, imprecision, indirectness, and publication bias) and the three criteria for uprating our confidence (large effect, dose–response gradient, and opposing confounding). Based on these criteria, the quality of evidence judgment can range from very low to high.

## Discussion

Controversy exists about the choice of drug, route of administration, and length of treatment in the therapy of neuroborreliosis. Evidence from RCTs on this topic are likely scarce; therefore, we will perform a comprehensive and systematic overview of the evidence incorporating RCTs and non-randomized studies examining pharmacological treatments for adults with neuroborreliosis. We will investigate for which intervention efficacy is reported. In this protocol, subgroup analyses and sensitivity analyses are predefined. Clinical important and much debated questions regarding differences in effects of varying antibiotic groups and length of antibiotic treatment will be investigated. Of particular interest is the adequate dose of doxycycline, which will be investigated separately. Adverse events of treatments will be evaluated, for which non-randomized studies are particularly valuable. Different to previous conducted reviews, another advantage will be the systematic assessment of risk of bias [16]. Our results will be important to clarify controversies and reduce uncertainty both for patients and healthcare providers. The summary and evaluation of the available body of evidence may lead to evidence based treatment recommendations for neuroborreliosis. Implications for future research can be drawn from the results.

## Appendix 1 Ovid MEDLINE search strategy

1. exp Lyme Disease/

2. lyme*.mp.

3. neuroborreliosis.mp.

4. borreli*.mp.

5. exp Borrelia/

6. (erythem* adj2 migran*).mp.

7. or/1-6

8. exp Brain/

9. brain*.mp.

10. mening*.mp.

11. spinal*.mp.

12. exp Nervous System Diseases/

13. encephal*.mp.

14. radiculi*.mp.

15. radiculo*.mp.

16. Facial Paralysis/

17. facial pal*.mp.

18. facial par*.mp.

19. Myelitis/

20. myel*.mp.

21. (nervous system adj5 dis*).mp.

22. neur*.mp.

23. polyneur*.mp.

24. polyradicul*.mp.

25. mononeur*.mp.

26. (nerve adj5 damage*).mp.

27. (nerve adj5 involvement).mp.

28. bannwarth*.mp.

29. vasculitis/

30. exp vasculitis, central nervous system/

31. vasculiti*.mp.

32. cranial nerve*.mp.

33. or/8-32

34. 7 and 33

## Appendix 2 SCOPUS search strategy

1. TITLE-ABS-KEY(lyme*) OR TITLE-ABS-KEY(neuroborreliosis) OR TITLE-ABS-KEY(borreli*) OR TITLE-ABS-KEY(erythema migrans)

2. TITLE-ABS-KEY(brain*) OR TITLE-ABS-KEY(mening*) OR TITLE-ABS-KEY(spinal*) OR TITLE-ABS-KEY(encephal*) OR TITLE-ABS-KEY(radiculi*) OR TITLE-ABS-KEY(radiculo*) OR TITLE-ABS-KEY(facial pal*) OR TITLE-ABS-KEY(facial par*) OR TITLE-ABS-KEY(myel*) OR TITLE-ABS-KEY(nervous system dis*) OR TITLE-ABS-KEY(neur*) OR TITLE-ABS-KEY(polyneur*) OR TITLE-ABS-KEY(polyradicul*) OR TITLE-ABS-KEY(mononeur*) OR TITLE-ABS-KEY(nerve AND damage*) OR TITLE-ABS-KEY(nerve AND involve*) OR TITLE-ABS-KEY(bannwarth*) OR TITLE-ABS-KEY(vasculiti*) OR TITLE-ABS-KEY(cranial nerve*)

3. 1 AND 2

## Appendix 3 CENTRAL search strategy

1. MeSH descriptor: [Borrelia] explode all trees

2. MeSH descriptor: [Lyme Disease] explode all trees

3. *borreli*

4. erythem* near/2 migran*

5. lyme*

6. 1 OR 2 OR 3 OR 4 OR 5

## Abbreviations

AAN: American Academy of Neurology; CSF: Cerebrospinal fluid; DGN: Deutsche Gesellschaft für Neurologie; EDSS: Expanded Disability Status Scale; EFNS: European Federation of Neurological Societies; IDSA: Infectious Disease Society of America; ILADS: International Lyme and Associated Diseases Society; NIS: Neurological Impairment Scale; NOS: Newcastle-Ottawa-Scale; RCT: Randomized-controlled trial; RoB: Risk of bias.

## Competing interests

SR reports receiving consulting and lecture fees, grant and research support from Bayer Vital GmbH, Biogen Idec, Merck Serono, Novartis, Sanofi-Aventis, Baxter, RG, and Teva. Furthermore, SR indicates that he is a founding executive board member of ravo Diagnostika GmbH. All other authors declare that they have no competing interests.

## Authors’ contributions

RD drafted the manuscript. JM and SR made substantial contributions to the conception and design. GR and SH made substantial contribution to the section on analysis of data. RD, MF, SS, HS, GR, SR, and JM revised the manuscript critically for important intellectual content. All authors read and approved the final manuscript.
